# Polymorphisms of the *IGF1R *gene and their genetic effects on chicken early growth and carcass traits

**DOI:** 10.1186/1471-2156-9-70

**Published:** 2008-11-07

**Authors:** Mingming Lei, Xia Peng, Min Zhou, Chenglong Luo, Qinghua Nie, Xiquan Zhang

**Affiliations:** 1 Department of Animal Genetics, Breeding and Reproduction, College of Animal Science, South China Agricultural University, Guangzhou 510642, Guangdong, PR China; 2 Institute of Animal Science, Guangdong Academy of Agricultural Science, Guangzhou 510640, Guangdong, PR China

## Abstract

**Background:**

The insulin-like growth factor I receptor (IGF1R) has an important effect on growth, carcass, and meat quality traits in many species. However, few studies on associations of the *IGF1R *gene with growth and carcass traits have been reported in chickens. The objectives of the present study were to study the associations of the *IGF1R *gene with chicken early growth and carcass traits using a neutral test, variation scan of the gene, genetic diversity, linkage disequilibrium and association analyses.

**Results:**

The tree generated from the amino acid sequences of 15 species showed that the *IGF1R *gene was conservative in the whole evolution among the mammalian animals and chickens. In a total of 10,818 bp of sequence, 70 single nucleotide polymorphisms were identified in the chicken *IGF1R *gene. The allelic and genotypic frequency distribution, genetic diversity and linkage disequilibrium of 18 single nucleotide polymorphisms (SNPs) in the Xinghua and White Recessive Rock chickens showed that six of them were possibly associated with growth traits. Association analyses showed that the A17299834G SNP was significantly associated with chicken carcass body weight, eviscerated weight with giblets, eviscerated weight, body weights at 28, 35, and 56 d of age, leg length at 56 d of age, and daily weight gain at 0–4 weeks. The haplotypes of the A17307750G and A17307494G were associated with early growth traits. The haplotypes of the A17299834G and C17293932T were significantly associated with most of the early growth traits and carcass traits.

**Conclusion:**

There were rich polymorphisms in the chicken *IGF1R *gene. Several SNPs associated with chicken early growth traits and carcass traits were identified in the *IGF1R *gene by genetic diversity, linkage disequilibrium, and association analyses in the present study.

## Background

The insulin-like growth factor 1 receptor (IGFIR) is a membrane glycoprotein mediating most biological actions of IGF-1 and IGF-2, which have an important effect on chicken growth, carcass, and meat quality traits [1-3]. Two receptors (IGF1R and IGF2R) were found in the mammals but only one (IGF1R) was found in the birds. IGF1R not only regulated the half-life time and activity of IGFs, but also played important roles on the key developmental stage and adult stage such as the cell life cycle, transplantation, metabolism, subsistence, proliferation, and differentiation.

Many variations in the genome affected gene expression at the transcription and translation levels [4,5]. Variations in the genes of somatotropic axis could function as candidates for the evaluation of their effects on animal growth and development traits. In humans, mutations at important regulatory sites of the *IGF1R *gene were associated with growth. Such mutations resulted in the failure of processing of proIGF1R to mature IGF1R and caused dysfunction and short stature of IGFR [6-9]. These variations affected partly the expression and physiological functions of the *IGF1R *gene, and subsequently affected growth. However, few studies on associations of the *IGF1R *gene with growth and carcass traits were reported in chickens.

In the present study, the objectives were to study the associations of the *IGF1R *gene polymorphism with chicken early growth and carcass traits. Polymorphisms of the chicken *IGF1R *gene were scanned in a 10,818 bp of sequence. The single nucleotide polymorphisms (SNPs) used in association analyses were selected based on the genetic diversity and linkage disequilibrium analyses in the Xinghua (XH) and White Recessive Rock (WRR) chickens. The associations of the SNPs or their haplotypes with chicken early growth and carcass traits were analyzed in a F_2_ resource population generated from a reciprocal cross between XH and WRR chickens.

## Results

### The molecular evolution of the *IGF1R *gene

**Figure 1:**
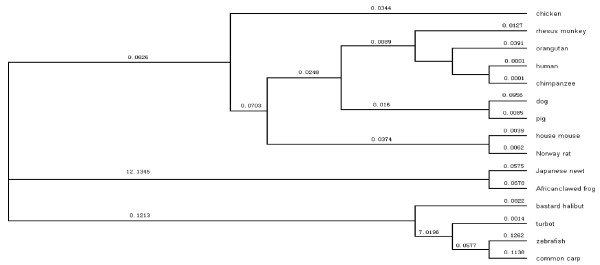
The ω value and UPGMA tree from CODEML arithmetic.

The tree generated from the amino acid sequences of 15 species showed that a positive selection was possible for the *IGF1R *gene in the early evolution of the Japanese firebelly newt and Africa clawed frog, and in the middle evolution of the zebrafish, turbot, and common carp (ω > 1). But the *IGF1R *gene was conservative in the whole evolution between the mammals and chickens (Figure 1).

### Variations of the chicken *IGF1R *gene

In the present study, 10,818 bp of sequence in the *IGF1R *gene, in which exon regions were preferred but segmental introns were also included (Table 1 in Supplementary Materials File 1), was scanned, and 70 SNPs were identified between XH and WRR chickens. Among the 70 SNPs, 7 SNPs were located in the 5' regulated region, 15 in the coding regions, 57 in the intron regions, and 1 in the 3' regulated region. Average density of SNPs was one SNP per 173 bp (70/12,038) in the whole region studied, with one SNP per 273 bp (15/4092 bp) in the coding regions, and one SNP per 140 bp (48/6726 bp) in the intron regions. Among the 70 SNPs, 51 SNPs are transition, 17 transversion, and 1 one base insertion/deletion. Fifteen SNPs were found in the coding regions, but only one SNP was non-synonymous mutation (Asn → Ser in the exon 3).

### Nucleotide diversity and neutral test

The nucleotide diversity of the chicken *IGF1R *gene was (2.87 ± 0.28) × 10^-3^, the nucleotide polymorphism was (2.62 ± 1.06) × 10^-3^, and all parameters of the neutral test were negative but not significant. For the 5' flanking region, the nucleotide diversity of the chicken *IGF1R *gene was (3.83 ± 1.62) × 10^-3^, the nucleotide polymorphism is (3.01 ± 1.0) × 10^-3^, all parameters of the neutral test were positive, and the values of Fu and Li's D, and F test were significant (P < 0.05). For the intron region, the nucleotide diversity of the chicken *IGF1R *gene was (3.52 ± 1.49) × 10^-3^, the nucleotide polymorphism was (3.98 ± 0.43) × 10^-3^, all parameters of the neutral test were negative but not significant. For the exon region, the nucleotide diversity of the chicken *IGF1R *gene was (1.59 ± 0.57) × 10^-3^, the nucleotide polymorphism was (1.43 ± 0.32) × 10^-3^, and all parameters of the neutral test were positive but not significant (Table 2 in Supplementary Materials File 1).

### Allelic frequency and heterozygosity in the XH and WRR chickens

Between the XH and WRR chickens, the allelic frequencies of C17393427T and A17323673G were significantly different at the P < 0.05 level and the allelic frequencies of the 7 SNPs, C17445985T, A17417734G, C17417042G, T17334342C, A17327275C, A17307494G, and A17299834G, were significantly different at the P < 0.01 level. No significant differences for mean heterozygosity of the 18 SNPs were found, but significant differences of A17313488G, A17307750G, A17307494G, A17299834G, and C17293932T were observed in the XH and WRR chickens (Table 3 in Supplementary Materials File 1).

### TagSNP of the *IGF1R *gene in the XH and WRR chickens

Eight TagSNP, C17293932T, A17299834G, A17307750G, A17313488G, C17393427T, T17416994C, G17445596A, and C17445985T, were identified in the XH chickens by HapBLOCK software. Another eight TagSNP, C17293932T, A17299834G, A17307494G, A17307750G, T17416994C, C17417042G, A17417734G and G17445596A, were also identified as TagSNPs in the WRR chickens.

### Linkage disequilibria of the *IGF1R *gene in the XH and WRR chickens

Average values of r^2 ^showed that the linkage disequilibria declined with increasing physical distance between SNP pairs in the XH and WRR chickens (Figure 1 in Supplementary Materials File 1). The effective extent of linkage disequilibrium was 27, 441 bp in the WRR chickens, but not found in the XH chickens. Possible regions of strong linkage disequilibrium were found between exon 6 and the 3' untranslating region (between A17327275C and C17293932T) in the WRR chickens (Table 4 in Supplementary Materials File 1).

### Association of the 6 SNPs with chicken early growth and carcass traits

Associations of the 6 SNP with chicken early growth and carcass traits were analyzed, but only the A17299834G of the chicken *IGF1R *gene was significantly associated with some growth and carcass traits. The A17299834G of the chicken *IGF1R *gene was significantly associated with chicken carcass weight, eviscerated weight with giblets, eviscerated weight, body weights at 28, 35, and 56 d of age, leg length at 56 d of age, and daily weight gain at 0–4 weeks (P < 0.05). Significantly and suggestively dominant effects of AG genotype were observed for chicken carcass weight, eviscerated weight with giblets, breast muscle weight, eviscerated weight, fat thickness under skin, fat width, body weights at 14, 21, 28, 35, 42, 49, 56, and 77 d of age, and leg length at 42 and 56 d of age (Table 1). In other words, the A17299834G SNP affected the chickens' early growth.

**Table 1:** Association of the A17299834G with chicken growth and carcass traits

**Traits**	**P**	**AA (24)**	**AG (87)**	**GG (327)**
*CW*	0.0138	1454 ± 49.61A	1378 ± 28.29A	1311 ± 14.40B
*Fat thickness under skin*	0.0811	4.71 ± 0.29a	4.31 ± 0.16ab	4.05 ± 0.08b
*Fat width*	0.0556	13.98 ± 0.84a	12.36 ± 0.48ab	11.84 ± 0.24b
*EWG*	0.0135	1334 ± 45.81A	1263 ± 26.12A	1201 ± 13.29B
*EW*	0.0165	1154 ± 40.71A	1095 ± 23.21A	1040 ± 11.81B
*Breast muscle weight*	0.0588	98.35 ± 3.72a	94.24 ± 2.12ab	89.86 ± 1.08b
*BW at 14 days*	0.0718	130.3 ± 3.42a	126.3 ± 1.95ab	122.6 ± 0.99b
*BW at 21 days*	0.1364	221.3 ± 6.51a	214.3 ± 3.73a	208.4 ± 1.89a
*BW at 28 days*	0.0313	320.1 ± 10.39a	319.5 ± 5.94a	305.7 ± 3.03a
*BW at 35 days*	0.0143	471.1 ± 15.87a	452.9 ± 9.16ab	428.1 ± 4.66b
*BW at 42 days*	0.2734	596.3 ± 21.50a	581.9 ± 12.29a	563.9 ± 6.24a
*LL at 42 days*	0.0608	62.45 ± 0.98a	61.38 ± 0.56ab	60.23 ± 0.28b
*BW at 49 days*	0.1730	744.4 ± 25.38a	719.1 ± 14.47a	697.4 ± 7.38a
*BW at 56 days*	0.0466	909.2 ± 30.27a	889.3 ± 17.36a	845.9 ± 8.78b
*LL at 56 days*	0.0066	74.58 ± 1.07A	74.26 ± 1.07A	71.48 ± 0.31B
*BW at 77days*	0.0829	1407 ± 59.32a	1363 ± 28.95a	1299 ± 14.66a
*DGW at 0–4 weeks*	0.0267	10.80 ± 0.37a	10.35 ± 0.21a	9.84 ± 0.10b

CW-carcass weight; EWG-eviscerated weight with giblets; EW-eviscerated weight

BW-body weight; LL-leg length; DGW daily weight gain

The capital letters indicate that multiple comparison is greatly significant (P < 0.01), and small letters indicate that multiple comparison is significant (P < 0.05).

### Haplotype structure within the 6 SNP in the F_2_ resource population

For the *IGF1R *gene, two haplotype blocks were observed in the F_2_ resource population. Block 1 comprised A17307750G and A17307494G, located between intron 17 and 18, and block 2 comprised A17299834G and C17293932T, located between exon 20 and the 3'untranslation region (Figure 2 in Supplementary Materials File 1).

In block 1, four haplotypes were observed in the F_2_ individuals of the resource population. Three distinct haplotypes, H1, H2, and H3, accounted for 95.4% of the total number of the four haplotypes. Among the four haplotypes, allele H4 had the lowest allelic frequency of 0.46%, and H1 had the highest allelic frequency of 51.60% (Table 5 in Supplementary Materials File 1). In block 2, four haplotypes were also observed in the F_2_ individuals of the resource population. Three distinct haplotypes, E1, E2, and E3, accounted for 96.6% of the four observed haplotypes. Among the four haplotypes, the allelic frequency of E4 was the lowest at 0.34%, the highest allelic frequency was 57.88% for haplotype E2.

### Associations of the haplotypes with chicken growth and carcass traits

Significant associations of the haplotypes of A17307750G and A17307494G with chicken growth and carcass traits were observed. The haplotypes of A17307750G and A17307494G were significantly associated with body weights at 28 and 49 d of age, and with daily weight gain at 0–4 weeks at the P < 0.05 level, and significantly associated with body weight at 35 d of age, and leg length at 42 and 49 d of age at the P < 0.01 level. The haplotypes composed of A17299834G and C17293932T affected the chickens' early growth. Significantly and suggestively dominant effects of H1H3 diplotype were observed for body weights at 7, 14, 21, 28 d of age, and daily weight gain at 0–4 weeks. The H2H4 diplotype was dominant for body weights at 35 and 49 d of age (Table 2). Significant associations of the haplotypes of A17299834G and C17293932T with chicken growth and carcass traits were observed. The haplotypes of A17299834G and C17293932T were significantly associated with breast angle width, eviscerated weight with giblets, breast muscle weight, body weights at 28 and 35 d of age, leg length at 56 d, and daily weight gain at 0–4 weeks (P < 0.05), and significantly associated with body weight at 35 d of age, and leg length at 42 and 49 d of age at the P < 0.01 level. In other words, the haplotypes affected the chickens' early growth. The E1E4 diplotype was dominant for body weights at 7, 14, 21, 28, 35, 42, and 49 d of age, daily weight gain at 0–4 weeks, carcass weight, eviscerated weight with giblets, eviscerated weight, breast muscle weight, brain neck weight, breast angle width, small intestines length at 90 d of age, and leg length at 56 d of age (Table 3). Multiple comparisons showed that the E1E4 diplotype had a dominant effect in the all traits.

**Table 2:** Association of the haplotype composed of A17307750G and A17307494G with chicken early growth and carcass traits

	**P**	**H1H1 (122)**	**H1H2 (184)**	**H1H3 (24)**	**H2H2 (91)**	**H2H3 (12)**	**H2H4 (4)**	**H3H3 (1)**
BW at 7 days	0.08	59.62 ± 0.69ab	60.38 ± 0.68ab	55.71 ± 1.77b	59.53 ± 0.90ab	53.91 ± 2.43b	55.97 ± 4.04ab	62.18 ± 7.18a
*BW at 14 days*	0.10	123.6 ± 1.49AB	125.5 ± 1.42AB	122.2 ± 3.69AB	123.1 ± 1.85AB	109.1 ± 5.20A	108.8 ± 7.82A	133.2 ± 15.65B
*BW at 21 days*	0.10	211.5 ± 2.85AB	211.9 ± 2.72AB	211.1 ± 7.00AB	209.4 ± 3.53AB	179.6 ± 9.86A	183.1 ± 14.82A	218.1 ± 29.66B
*BW at 28 days*	0.03	310.2 ± 4.54AB	308.8 ± 4.36AB	322.5 ± 11.26AB	309.9 ± 5.62AB	256.2 ± 15.9A	296.8 ± 23.77AB	347.3 ± 47.13B
*BW at 35 days*	0.005	435.8 ± 6.91AB	437.1 ± 6.61AB	442.0 ± 17.41AB	436.2 ± 8.56AB	344.8 ± 24.74A	465.2 ± 36.33B	455.1 ± 72.08B
*BW at 42 days*	0.102	578.5 ± 9.37AB	567.1 ± 8.99AB	594.1 ± 23.09B	561.7 ± 11.63AB	473.2 ± 32.45A	543.6 ± 48.65AB	571.9 ± 97.40AB
*LL at 42 days*	0.008	60.70 ± 0.42AB	60.32 ± 0.40AB	61.91 ± 1.04AB	60.67 ± 0.52AB	55.45 ± 1.46A	63.75 ± 2.19B	60.93 ± 4.39AB
*LD at 42 days*	0.09	7.89 ± 0.06AB	7.86 ± 0.06AB	7.84 ± 0.16AB	7.86 ± 0.08AB	7.19 ± 0.23A	7.93 ± 0.35AB	8.73 ± 0.71B
*BW at 7 days*	0.04	712.9 ± 11.02AB	703.7 ± 10.52AB	718.9 ± 27.45AB	697.3 ± 13.71AB	606.9 ± 38.94A	778.8 ± 58.15B	646.2 ± 115.44AB
*LL at 7 days*	0.001	67.91 ± 0.61AB	67.71 ± 0.68AB	66.98 ± 1.65AB	67.93 ± 0.71AB	59.68 ± 2.23A	73.18 ± 2.75B	67.68 ± 4.80AB
*LD at 7 days*	0.09	9.28 ± 0.09A	9.30 ± 0.08A	9.64 ± 0.20A	9.48 ± 0.11A	9.26 ± 0.33A	9.29 ± 0.43A	11.53 ± 0.82B
*DGW at 0–4 weeks*	0.04	10.00 ± 0.16A	9.96 ± 0.15A	10.40 ± 0.41A	10.02 ± 0.20A	8.12 ± 0.57A	9.56 ± 0.84AB	11.23 ± 1.68B

BW – body weight, LL-leg length, LD-leg diameter, DGW-daily weight gain

Capital letters indicate that multiple comparison is greatly significant (P < 0.01), and small letters indicate that multiple comparison is significant (P < 0.05).

**Table 3:** Association of the haplotype composed of SNP A17299834G and C17293932T with chicken growth and carcass traits

**Traits**	**P**	**E1E1 (55)**	**E1E2 (107)**	**E1E3 (14)**	**E1E4 (3)**	**E2E2 (164)**	**E2E3 (70)**	**E3E3 (27)**
*Breast angle width*	0.022	60.23 ± 0.85	60.74 ± 0.54	64.38 ± 1.41	67.86 ± 2.86	59.81 ± 0.42	60.32 ± 0.75	61.36 ± 1.15
*CW*	0.061	1353 ± 38.55	1302 ± 24.46	1453 ± 64.03	1481 ± 129.10	1301 ± 19.36	1351 ± 34.23	1434 ± 51.93
*EWG*	0.043	1245 ± 35.56	1245 ± 35.56	1333 ± 59.07	1373 ± 119.09	1193 ± 17.86	1238 ± 31.57	1316 ± 47.91
*EW*	0.062	1080 ± 31.62	1032 ± 20.07	1151 ± 52.53	1182 ± 105.90	1030 ± 15.88	1072 ± 28.08	1136 ± 42.60
*Breast muscle weight*	0.034	93.48 ± 2.87	89.20 ± 1.82	104.00 ± 4.77	108.23 ± 9.63	88.74 ± 1.44	90.72 ± 2.55	95.79 ± 3.87
*Brain and neck weight*	0.063	128.3 ± 4.40	116.4 ± 2.79	134.7 ± 7.31	141.1 ± 14.73	122.9 ± 2.21	124.0 ± 3.91	129.0 ± 5.92
*Small intestine length*	0.101	139.2 ± 2.41	136.6 ± 1.53	145.0 ± 4.00	147.9 ± 8.08	139.5 ± 1.21	135.1 ± 2.14	136.2 ± 3.25
*BW at 14 days*	0.071	127.8 ± 2.65	121.6 ± 1.68	127.8 ± 4.40	138.2 ± 8.88	121.6 ± 1.33	124.8 ± 2.36	129.1 ± 3.57
*BW at 21 days*	0.134	215.1 ± 5.10	206.6 ± 3.22	219.7 ± 8.38	251.0 ± 19.73	207.1 ± 2.55	211.0 ± 4.49	218.9 ± 6.79
*BW at 28 days*	0.032	315.5 ± 8.10	303.9 ± 5.09	333.7 ± 13.33	374.4 ± 26.78	302.9 ± 4.05	312.0 ± 7.15	326.5 ± 10.83
*BW at 35 days*	0.023	439.2 ± 12.34	424.8 ± 7.97	486.4 ± 20.59	521.0 ± 41.10	425.0 ± 6.25	440.1 ± 11.15	462.1 ± 16.60
*Leg length at 56 days*	0.024	72.03 ± 0.83	71.10 ± 0.53	74.97 ± 1.38	77.12 ± 2.79	71.42 ± 0.41	72.77 ± 0.74	74.21 ± 1.12
*DGW at 0–4 weeks*	0.021	10.24 ± 0.29	9.76 ± 0.18	10.85 ± 0.47	12.30 ± 0.95	9.74 ± 0.14	10.08 ± 0.25	10.60 ± 0.38

CW-carcass weight; EWG-eviscerated weight with giblets; EW-eviscerated weight; BW-body weight;

DGW daily weight gain.

Capital letters indicate that multiple comparison is greatly significant (P < 0.01), and small letters indicate that multiple comparison is significant (P < 0.05).

## Discussion

A molecular phylogenetic tree generated from the amino acid sequences of 15 species showed that the *IGF1R *gene was conservative and favored subsistence in the evolution of these species. At the same time, the ω value in the mammals and chickens proved that the *IGF1R *gene was conservative in the whole evolution of the mammal animals and chickens. Analysis of evolutionary conservation had provided insights into essential regions of molecules such as IGF-I and their receptors, in which the tyrosine kinase domain is highly conserved [10]. Sequence comparison showed that the primary structures of zebrafish IGF1R have been highly conserved in vertebrates [11]. However, the nucleotide diversity of the chicken *IGF1R *gene seems to be much higher, and chickens have a higher SNP incidence and polymorphisms.

Among the 70 SNPs, 18 SNPs were selected to study the allelic frequencies, heterozygosity, TagSNP, and linkage disequilibria of the XH and WRR chickens based on their location, possible transcriptional site and the distribution density. In 9 of the 18 SNPs, there were significant differences of allelic frequencies between the XH and WRR chickens. These SNPs with obviously different allelic frequencies between slow-growing XH and fast-growing WRR chickens could contribute to their divergent growth performance. Considering some associations might be false positives, allelic frequency differences between XH and WRR may partially provide support to the results of the association analyses.

SNP markers were preferred for disease association studies because of their high abundance along the human genome, the low mutation rate, and accessibility to high-throughput genotyping. Thus, the selection of a maximally informative set of SNPs (tag SNPs) for genome-wide association studies has recently attracted much attention. In those high LD regions, only a small number of SNPs were sufficient to capture most of haplotype structure [12]. In the present study, 8 different TagSNP were found in the XH and WRR chickens.

The purpose of the study was to find functional SNPs by genetic diversity, linkage disequilibrium and association analyses of the SNPs with the economically important traits. There were several successful examples in plants and humans. Yu identified 6 SNPs of the *rabl7 *gene associated with drought tolerance in maize based on genetic diversity and linkage disequilibrium [13]. Fu et al. reported a systematic search for polymorphisms in the *CASQ1 *gene on chromosome 1q21 and identified a significant association between the *CASQ1 *polymorphism and type 2 diabete by linkage disequilibrium for the first time [14]. Using linkage disequilibrium, some important SNPs or QTL were found [15-17]. Morahan et al. reported that a single base change in the 3'UTR showed a strong linkage disequilibrium with the T1D susceptibility locus and the alleles showed different levels of expression in cell lines [18]. In the present study, a possible strong linkage disequilibrium region was found in the WRR chickens. The results showed that some SNPs were linked and were tightly scattered between the C17293932T and A17327275C. Combining the results of the linkage disequilibrium, 6 SNPs, C17293932T, A17299834G, A17307494G, A17307750G, G17445596A, and C17445985T, were selected and used in the association analyses.

The results in the present study showed that the *IGF1R *gene affected the chickens' early growth, which is consistent with reports on humans. Previous studies on humans indicated that there were important associations of the *IGF1R *gene with growth and development [6,7,9]. Kawashima et al. also reported that a heterozygous mutation (R709Q) changing the cleavage site from Arg-Lys-Arg-Arg to Arg-Lys-Gln-Arg was identified in a 6-year-old Japanese girl and the mutation resulted in the failure of processing of the IGF1R proreceptor to mature IGF1R, causing short stature [8]. Mutations or SNPs in the *IGF1R *gene could partially affect the gene expression, and thus could affect animal physiological metabolism and growth. Other studies proved that the association of haplotype with economic traits was more predominant and reliable [19-21], perhaps due to the multiple-loci interaction of the haplotype.

The chicken *IGF1R *gene was located on GGA10 with a physical distance around 187 Mb, and a genetic distance close to 100 cM. Recently, some quantitative trait loci (QTLs) associated with growth and carcass traits were found in the GGA10 [22,23]. Rabie et al. showed that a single QTL related to body weight at 5 weeks under ascites conditions was located on 82–101 cM [24]. Zhou et al. reported that 5 QTLs were identified to be associated with abdominal fat weight, body wight, heart weight, liver weight, spleen weight at the 11–120 cM [25]. These studies suggest that the associations of the SNP or haplotype with economic traits in the present study were reliable.

In conclusion, there were rich polymorphisms in the chicken *IGF1R *gene. Several SNPs associated with chicken early growth traits and carcass traits were identified in the *IGF1R *gene by genetic diversity, linkage disequilibrium, and association analyses in the present study.

## Materials and methods

### DNA pools

The initial SNP discovery was carried out on the DNA pools of 7 breeds, XH chickens, Taihe Silkie chickens, Beijing Fatty chickens, Yangshan chickens, Dwarf chickens, White Leghorn chickens, and WRR chickens. Ten individuals for each breed generated one pooled DNA sample. An equal amount of DNA was taken from each individual and was pooled to generate the 7 pooled DNA samples. DNA samples were diluted and reassessed to obtain an equal amount of DNA from each individual.

### A F_2 _resource population for association analyses

A F_2 _resource population was constructed by reciprocal crossing the XH with WRR chickens [26]. The F_2 _individuals were raised in floor pens and fed commercial corn-soybean diets that met NRC requirements. The birds from six batches were kept in different pens, and the sizes of all pens were the same. The body weight was measured in grams at hatch, 7, 14, 21, 28, 35, 42, 49, 56, and 90 d of age. The 434 individuals from the F_2 _generations (221 male and 213 female) were slaughtered at 90 d of age. Shank length (mm), head width (mm), breast width (mm), breast depth (mm), body length (cm), breast angle width (degree), carcass weight (g), fat thickness under skin (mm), fat width (mm), eviscerated weight with giblets (g), eviscerated weight (g), breast muscle weight (g), leg muscle weight (g), wing weight (g), abdominal fat pad weight (g), head and neck weight (g), weights of heart, liver, and gizzard (g), and small intestine length (cm) were recorded.

### Chicken populations for genetic diversity study and linkage disequilibrium analyses

Two unrelated populations, consisting of 112 XH individuals and 86 WRR individuals, respectively, were sampled for genetic diversity investigation in the present study. The XH and WRR chickens were parents of the F_2 _resource population, both from Guangdong Wens Foodstuff Corporation Ltd. (Guangdong, China). The XH chicken is a Chinese native breed with slow growth rate, and the WRR chicken is of fast growth rate. There is significant difference in growth and carcass traits between the XH and WRR chickens.

### Primer design for sequencing

Available sequences of the chicken *IGF1R *gene were used as templates for designing specific primers by the Genetool software . Twenty-two primers were obtained and an optimal length of the PCR product was set between 450 and 800 bp. Exon regions were preferred, but segmental intron sequences were also included. Details were listed in Table 1 in Supplementary Materials File 1.

### Selections of the SNPs used for genetic diversity investigation and linkage disequilibrium analyses

For genetic diversity investigation and linkage disequilibrium analyses, 18 SNPs (Table 3 in Supplementary Materials File 1) were selected based on the following criterions. (1) Positions: SNP in coding regions were selected preferably over those from non-coding regions. (2) Functional domain: SNP in the important structural and conservative functional domains such as extracellular domain, joint of α subunit and β subunit, and tyrosine kinase domain, were preferred to those in the other domains. (3) Potential regulating units: SNP located at the potential regulatory sites of the un-translating region were preferred to those located at the other sites. (4) Density: An average density of 1 SNP per 8.3 kb was determined, and a total of 18 SNPs were selected in a 150 kb full sequence of the chicken *IGF1R *gene.

### Neutral test of the *IGF1R *gene

The ω value was calculated using codeml program of PAML software [27], and formula was followed,

ω = dN/dS,

Where dN and dS are the number of non-synonymous substitutions per non-synonymous site, and the number of synonymous substitutions per synonymous site, respectively. In the present study, the selection function of the DNA sequences was analyzed in species using Branch model of codeml program (M0 and M1), and the selection function of the amino acid was analyzed using site model (M7 and M8) in the evolution .

### Discovery and identification of SNPs

SNPs having different allelic frequencies in the 7 pooled DNA samples were validated by sequencing the PCR product from both ends and by re-sequencing PCR products. SNPs were identified by alignment of sequences using the BioEdit program . SNP calls were made on sequences of high quality.

### Amplification and Genotyping

The PCR was performed in a final volume of 25 μL containing 1 μL genomic DNA (2.5 ng/μL), 0.25 μL each primer (25 μM), 0.5 μL deoxynucleotide triphosphates (10 μM) mixture, 1.5 μL MgCl_2 _(25 mM), 0.2 μL DNA polymerase (5 U/μL) (TaKaRa, Japan) and 2.5 μL 10 × reaction buffer on an ABI 2700 thermal cycle1 with the following profile, initial denaturation at 94°C for 4 min; 35 cycles of 94°C for 30 s, 60°C for 30 s, and 72°C for 30 s, and a final elongation at 72°C for 5 min.

### Nucleotide diversity of the chicken *IGF1R *gene

We used DNASP4.10 to perform tests of neutrality on the basis of the allelic frequency spectrum [28]. These included Tajima's D [29], and Fu and Li's D and F test statistics [30].

### Tag SNP Selection

In the dynamic programming algorithm for haplotype block partitioning and tag SNP selection based on haplotype data, Zhang et al. used the following recursive formula [19,31],

Sj = min{Si_ 1 + f(i,..., j),

if block(i,..., j) = 1}(1 _ j < n),

Where f(i,..., j) is the number of tag SNPs in this block, block(i,..., j) is a Boolean function, and block(i,..., j) = 1 if and only if SNP (i,..., j) can form a block, Sj is the minimum number of tag SNPs for the optimal haplotype block partition of the first j SNPs, and S0 = 0 .

### Statistical Analyses

The difference of allelic frequencies between the two unrelated chicken populations was tested using Mantel-Haenszel ChiSquare (SAS 8.1 FREQ).

The linkage disequilibrium r^2 ^value between each pair of SNPs and the haplotype structure of SNP within the gene were estimated by Haploview [32].

Haplotypes were constructed based on haplotype structure of the 18 SNPs in all 434 experimental animals by use of the PHASE 2.0 programme [33], whose function was to reconstruct haplotypes from the population data.

Data were analyzed by the GLM procedure of SAS 8.0 (Statistical Analysis Systems Institute Inc., Cary, NC) and the genetic effects were analyzed by a mixed procedure according to the following model,

Y= *μ *+*S*_*i*_+*B*_*j*_+*g*_*k*_+*f*_*x*_+*e*_*ijkx*_

Where Y represented the dependent variable, *μ*, *S*_*i*_, *B*_*j*_, *g*_*k*_, *f*_*x*_, and *e*_*ijkx *_represented the population mean, fixed effects of sex, fixed effects of hatch, genotype effect, family effect, and random error, respectively. Multiple comparisons were analyzed with least squares means, followed by the multiple comparison procedure, the multiple comparison procedures was followed:

$$\begin{array}{l} Y_{i}-{\bar{Y}}_{i}=(Y_{i}-{\hat{Y}}_{i})+({\hat{Y}}_{i}-{\bar{Y}}_{i})\\ \text{Where }\sum {\left(Y_{i}-{\hat{Y}}_{i}\right)}^{2}\text{ was least value},\text{ and }\sum {\left(Y_{i}-{\hat{Y}}_{i}\right)}^{2}=0 \end{array}$$

## Abbreviations

Bp: basepair; IGF1R: insulin-like growth factor I receptor; QTL: quantitative trait loci; SNPs: single nucleotide polymorphisms; UTR: un-translating region; WRR: White Recessive Rock chicken; XH: Xinghua chicken.

## Authors' contributions

ML contributed to the genotyping of most of the SNPs, summarized the data and drafted the manuscript. XP contributed to the genotyping of 6 SNPs in XH and WRR chickens. MZ contributed to the genotyping of 5 SNPs in the XH and WRR chickens. CL contributed to linkage disequilibrium analyses and haplotype construction. QN contributed to the design of the study and the revision of this manuscript. XZ designed the study, supervised the study, edited and made final improvements of this manuscript.

## Supplementary Material

Additional file 1 **Linkage disequilibrium of the Xinghua chickens and Recessive White Rock chickens.** Pairwise LD versus physical distance between all pairwise SNP, average values of r2 show that LD declines with increasing physical distance between SNP pairs. Click here for file
