# Supplementary material for: Polymorphisms of the IGF1R gene and their genetic effects on chicken early growth and carcass traits
Source: BMC Genet. 2008 Nov 7;9:70. doi: 10.1186/1471-2156-9-70 (PMC2628351; doi:10.1186/1471-2156-9-70)
Supplement: Additional file 1 — Linkage disequilibrium of the Xinghua chickens and Recessive White Rock chickens. Pairwise LD versus physical distance between all pairwise SNP, average values of r2 show that LD declines with increasing physical distance between SNP pairs. [file 1471-2156-9-70-S1.doc]

# Supplementary Materials

**Figure legends**

**Figure 1**. Linkage disequilibrium of the Xinghua chickens and Recessive White Rock chickens. Pairwise LD versus physical distance between all pairwise SNP, average values of r^2^ show that LD declines with increasing physical distance between SNP pairs.

**Figure 2.** Haplotype structure within 6 polymorphic sites in the F_2_ resource population

**Figure 1**


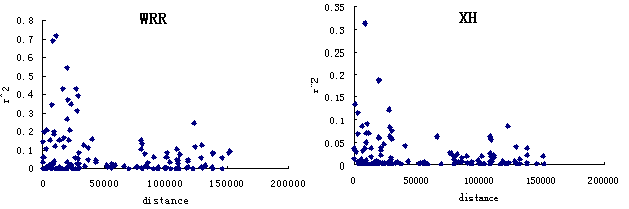


**Figure 2**


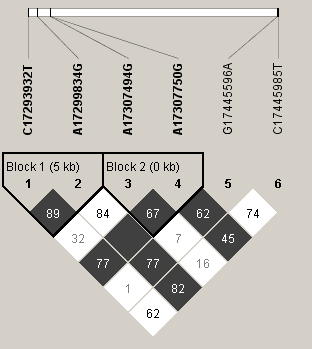


**Tables**

**Table 1. Primer sequences for SNP identification**

| Primer | Primer sequences |
| --- | --- |
| 501 | F: 5'CAGCGTCAACAAGGAAATAGAT 3', R: 5'GCTTTCAGTCCCTCCAGTCATA 3' |
| 511 | F: 5'GCCCTCCCAGTTCTGTGTGTG 3', R: 5'CGCGACGCCGAACAAATT 3' |
| 512-1 | F: 5'GCTGCTGGTAGTGCTCGTAGT 3', R: 5'CGGGTTATGTTCCTCAAGTTGT 3' |
| 512-2 | F: 5'TGCCTTGGTGATCTTCGAAAT 3', R: 5'GGGCCTACATACCAGAATAAAAG 3' |
| 513 | F: 5'GAGCCCAGGCAATGAAGACA 3', R: 5'CCAAATCACATCTTCCACCAAAT 3', |
| 514 | F: 5'TGGGTTGATGCATTTCCTTAAG 3', R: 5'GCCGACTACCTATTTCATCTGC 3' |
| 515 | F: 5'GCCATCTGTTAACTGCCACTTC 3', R: 5'TTCCAGTTGCAATCCCCTCAT 3' |
| 516 | F: 5'CTTGGTGGGAATTGGTGATG 3', R: 5'GCACAAAACAATCAAGCAGAAT 3' |
| 517 | F: 5'GGCACTGCTGGAAAGACTAAT 3', R: 5'CCCAACTCATTTTCCCAACT 3' |
| 518 | F: 5'GGTGGAAGGCTGAGGTGAGACA 3 ', R: 5'TTGGGAAGAGGAGTTGGATGCT 3' |
| 519 | F: 5'GCCAGTACCATCCATTCCCTTAG 3', R: 5'TGGGACGAAGATCTGCAATTAAG 3' |
| 520 | F: 5'TGCTTTTTCACCAATTTTCTT 3', R: 5'TCCCTGTTTTCAAAGATCTGTC 3' |
| 521 | F: 5'CCCCCACCATTAACTGTTG 3', R: 5'GGCAACAAGACTAATAGGGAACT 3' |
| 522 | F: 5'GCAGTGTCTCATTCCTTTGTCTT 3', R: 5'CTGGCTGCACCTCATATACCT 3' |
| 523 | F: 5'GCATGTGAAACCAAACTCCAGAC 3', R: 5'TCCCAAGCAGCAGGCAGATAC 3' |
| 524 | F: 5'GCAGCTGAAAGTACCGTGTCT 3', R: 5'CAGGCAGACAACATTCACATAC 3' |
| 525 | F: 5'CCTTTGGGATTGGTTAGTTTCA 3', R: 5'TGCCGAGTTGAAGCTAAAATC 3' |
| 526 | F: 5'GGCGGGAGGGTGTTCATG 3', R: 5'TGCGGGAAACAGGACTTGATAC 3' |
| 527 | F: 5'GCCCATAGCCTACGAAAGAGC 3', R: 5'TGCTCACACGTCTTAAATCACAG 3' |
| 528 | F: 5'CGTGGTAAAAATGCCTGCT 3', R: 5'CGCAGTGGATAACGAGAGC 3' |
| 529 | F: 5'CTGGTCAGCGCAAGGAGAT 3', R: 5'GGCAGACAGGGCAGAAACAG 3' |
| 530 | F: 5'GGGCCTGTAAAAATTCTGTCAC 3', R: 5'TGCGTATGTTTCTGTCAGGTTCT 3' |
| 531 | F: 5'CCAGCTTCGAGGAGAGACAG 3', R: 5'CACCTGGCCTGCGATATCTTT 3' |

**Table 2. The chicken *IGF1R*** gene polymorphisms

|  | Polymorphisms | | | | | | Neutrality tests | | |
| --- | --- | --- | --- | --- | --- | --- | --- | --- | --- |
|  | *L* | *S* | *H* | *Л* (×10^-3^) | *Θ* (×10^-3^) | *R_M_* | *D_T_* | *D_FL_* | *F_FL_* |
| Full sequences | 12038 | 70 | 15 | 2.62 ± 1.06 | 2.87 ± 0.28 | 30 | -0.3823 | -0.6011 | -0.6224 |
| 5' UTR | 1220 | 7 | 7 | 3.83 ± 1.62 | 3.01 ± 1.0 | 2 | 1.4370 | 1.5330^*^ | 1.6554^*^ |
| Intron | 6726 | 48 | 15 | 3.52 ± 1.49 | 3.98 ± 0.43 | 25 | -0.5063 | -0.79074 | -0.8198 |
| Exon | 4092 | 15 | 15 | 1.59 ± 0.57 | 1.43 ± 0.32 | 8 | 0.4617 | 0.0646 | 0.2000 |

*L*, sequence length in base pairs; *S*, number of segregating sites; π, average difference per base pair between two randomly chosen sequences; *θ_W_,* Watterson’s estimate of *θ* per site; *H*, number of haplotypes; *R_M_*, minimum number of recombination events; *D_T_*, Tajima’s D; *D_FL_*, Fu and Li’s D; *F_FL_*, Fu and Li’s F; *P < 0.05; **P < 0.01

**Table 3. Eighteen SNP allelic frequencies, ChiSquare test and heterozygosity in the XH and WRR** **chickens**

| Locus | Allelic frequencies | | ChiSquare | Heterozygosity | |
| --- | --- | --- | --- | --- | --- |
|  | XH | WRR | XH-WRR | XH | WRR |
| C17445985T | 0.62 | 0.43 | 9.59** | 0.467 | 0.500 |
| G17445596A | 0.82 | 0.42 | 1.01 | 0.293 | 0.362 |
| G17440010A | 0.00* | 0.00* | 0 | 0.000 | 0.000 |
| A17417734G | 0.57 | 0.59 | 13.27** | 0.488 | 0.494 |
| T17416994C | 0.55 | 0.31 | 0.32 | 0.488 | 0.499 |
| C17393427T | 0.99 | 0.35 | 11.61** | 0.304 | 0.012 |
| C17417042G | 0.36 | 0.78 | 8.27* | 0.467 | 0.307 |
| A17337024G/C | 0.13, 0.35 | 0.00, 0.99 | 6.02 | 0.605 | 0.594 |
| T17334342C | 0.51 | 0.31 | 24.14** | 0.497 | 0.392 |
| A17327275C | 0.54 | 0.48 | 21.37** | 0.494 | 0.444 |
| A17323673G | 0.54 | 0.04 | 6.18* | 0.500 | 0.503 |
| T17317101C | 0.50 | 0.93 | 2.3 | 0.501 | 0.500 |
| A17316026T | 0.64 | 0.82 | 5.56 | 0.459 | 0.472 |
| A17313488G | 74.26 | 0.41 | 0.61 | 0.383 | 0.444 |
| A17307750G | 0.87 | 0.99 | 4.71 | 0.218 | 0.269 |
| A17307494G | 0.46 | 0.99 | 9.26** | 0.496 | 0.495 |
| A17299834G | 0.81 | 0.97 | 14.39** | 0.326 | 0.475 |
| C17293932T | 0.80 | 0.99 | 4.35 | 0.338 | 0.459 |
| Mean |  |  |  | 0.407 ± 0.141 | 0.401 ± 0.163 |

**Table 4. Linkage disequilibrium of SNP in the WRR chickens**

| SNP | 1 | 2 | 3 | 4 | 5 | 6 | 7 | 8 | 9 | 10 | 11 | 12 | 13 | 14 | 15 | 16 | 17 |
| --- | --- | --- | --- | --- | --- | --- | --- | --- | --- | --- | --- | --- | --- | --- | --- | --- | --- |
| 1 |  |  |  |  |  |  |  |  |  |  |  |  |  |  |  |  |  |
| 2 | 0.156 |  |  |  |  |  |  |  |  |  |  |  |  |  |  |  |  |
| 3 | 0.155 | 0.345 |  |  |  |  |  |  |  |  |  |  |  |  |  |  |  |
| 4 | 0.001 | 0.028 | 0.038 |  |  |  |  |  |  |  |  |  |  |  |  |  |  |
| 5 | 0.268 | 0.002 | 0.016 | 0.017 |  |  |  |  |  |  |  |  |  |  |  |  |  |
| 6 | 0.209 | 0.434 | 0.692 | 0.024 | 0.01 |  |  |  |  |  |  |  |  |  |  |  |  |
| 7 | 0.351 | 0.169 | 0.185 | 0.04 | 0.209 | 0.2 |  |  |  |  |  |  |  |  |  |  |  |
| 8 | 0 | 0 | 0 | 0 | 0 | 0 | 0 |  |  |  |  |  |  |  |  |  |  |
| 9 | 0.126 | 0.433 | 0.547 | 0.036 | 0.007 | 0.717 | 0.124 | 0 |  |  |  |  |  |  |  |  |  |
| 10 | 0.16 | 0.012 | 0 | 0.061 | 0.372 | 0.002 | 0.118 | 0 | 0.005 |  |  |  |  |  |  |  |  |
| 11 | 0.037 | 0.115 | 0.086 | 0.008 | 0.021 | 0.154 | 0 | 0 | 0.198 | 0.107 |  |  |  |  |  |  |  |
| 12 | 0.044 | 0.052 | 0.026 | 0.001 | 0.01 | 0.027 | 0.007 | 0 | 0.041 | 0 | 0.147 |  |  |  |  |  |  |
| 13 | 0.003 | 0.01 | 0.008 | 0.031 | 0.003 | 0.01 | 0.007 | 0 | 0.012 | 0.016 | 0.005 | 0.026 |  |  |  |  |  |
| 14 | 0.247 | 0.005 | 0 | 0.049 | 0.035 | 0.005 | 0.006 | 0 | 0.001 | 0.084 | 0.103 | 0.155 | 0.007 |  |  |  |  |
| 15 | 0.12 | 0.041 | 0.029 | 0.046 | 0.002 | 0.086 | 0.065 | 0 | 0.063 | 0.014 | 0.001 | 0.138 | 0.008 | 0.001 |  |  |  |
| 16 | 0.086 | 0.06 | 0.085 | 0.007 | 0.018 | 0.128 | 0.06 | 0 | 0.051 | 0 | 0 | 0.001 | 0.02 | 0.056 | 0.314 |  |  |
| 17 | 0.097 | 0 | 0.001 | 0.083 | 0.014 | 0.006 | 0.009 | 0 | 0.011 | 0.079 | 0.105 | 0.048 | 0.007 | 0.396 | 0.027 | 0.062 |  |

1, 2, 3, 4, 5, 6, 7, 8, 9, 10, 11, 12, 13, 14, 15, 16, and 17 indicate C17293932T, A17299834G, A17307494G, A17307750G, A17313488G, A17316026T, T17317101C, A17323673G, A17327275C, T17334342C, A17337024G, C17337042G/C, 17393427T, T17416994C, A17417734G, G17445596A, and C17445985T.

**Table 5. Haplotype of the chicken *IGF1R* gene in the F_2_** of the resource population

| SNP | Name | Haplotype | | Number | Frequency |
| --- | --- | --- | --- | --- | --- |
|  |  | C17337042G | A17337024C/G |  |  |
| C17337042G and A17337024C/G | B1 | C | A | 2 | 0.23 |
|  | B2 | C | C | 281 | 32.08 |
|  | B3 | G | A | 101 | 11.53 |
|  | B4 | G | C | 492 | 56.16 |
| A17307750G and A17307494G | H1 | A | A | 452 | 51.60 |
|  | H2 | A | G | 382 | 43.61 |
|  | H3 | G | A | 38 | 4.34 |
|  | H4 | G | G | 4 | 0.46 |
| A17299834G and C17293932T | E1 | G | C | 234 | 26.71 |
|  | E2 | G | T | 507 | 57.88 |
|  | E3 | A | T | 132 | 15.07 |
|  | E4 | A | C | 3 | 0.34 |
